# Disseminate Cutaneous Vasculitis Associated with Durvalumab Treatment—Case Report, Mini-Review on Cutaneous Side Effects of Immune Checkpoint Inhibitor Therapies with Machine Learning Perspectives

**DOI:** 10.3390/life14091062

**Published:** 2024-08-25

**Authors:** Gyula Laszlo Fekete, Laszlo Barna Iantovics, Júlia Edit Fekete, Laszlo Fekete

**Affiliations:** 1 Department of Dermatology, George Emil Palade University of Medicine, Pharmacy, Science and Technology of Targu Mures, 540142 Targu Mures, Romania; 2 Department of Electrical Engineering and Information Technology, George Emil Palade University of Medicine, Pharmacy, Science and Technology of Targu Mures, 540142 Targu Mures, Romania; 3 National Institute of Public Health, Regional Center for Public Health, 540142 Targu Mures, Romania; 4 CMI Dermamed Private Medical Office, 540530 Targu Mures, Romania; 5 Doctoral School, George Emil Palade University of Medicine, Pharmacy, Science and Technology of Targu Mures, 540142 Targu Mures, Romania

**Keywords:** small-cell lung cancer, cancer, immunotherapy, durvalumab, vasculitis, cutaneous side effect, artificial intelligence, machine learning, intelligent agent

## Abstract

Durvalumab is an IgG1 monoclonal antibody that has efficacy in many advanced-stage cancers, especially in small-cell lung cancer. The efficacy of durvalumab can be enhanced by chemotherapy. Cutaneous side effects due to treatment with durvalumab are usually self-limiting and easily manageable. We present a clinical case of a female patient aged 61, with small-cell lung carcinoma in stage III B, cT3N2M, who developed a disseminated cutaneous vasculitis after seven months of durvalumab monotherapy, having previously been treated with polychemotherapy according to oncological protocols. To the best of our knowledge, based on a comprehensive search in leading databases, like Web of Science, Scopus, PubMed and some others, ours is the first published case of disseminated cutaneous vasculitis as a result of Durvalumab treatment. Anticancer immunotherapy targeting immune checkpoint inhibition (ICI) has transformed the treatment and evolution of patients with multiple varieties of hematologic cancers. In this context, the cutaneous side effects due to immune checkpoint inhibitor therapies are very few in the scientific literature. Based on this need, we have performed a mini-review of cutaneous side effects due to immune checkpoint inhibitor therapies that treat actual aspects in this sense. We also present some artificial intelligence challenges and future perspectives in the combination of human reasoning and reasoning based on Artificial Intelligence for study of the very rare Disseminate cutaneous vasculitis associated with Durvalumab treatment.

## 1. Introduction

Anticancer immunotherapy targeting immune checkpoint inhibition (ICI) has transformed the treatment and evolution of patients with different types of solid and hematologic tumors. The most significant improvements have been documented in patients with melanoma, non-small-cell lung, bladder, renal, urothelial, cervical, and colorectal cancers, Merkel cell carcinoma, and Hodgkin lymphoma [1]. Immune checkpoint inhibitors (ICIs) are used as immunotherapeutic applications for oncologic patients with compromised immune systems and can upgrade the capacity of T-cells to destroy cancer cells. Their efficiency may differ at different ages, especially in older patients. Their lower efficacy in elderly patients is unexplained, though the hypothesis of immuno-senescence and inflammation due to age-related changes in the immune system has been suggested [2,3]. Monoclonal antibodies (mAbs) which target immune checkpoints (i.e., anti-CTLA: ipilimumab; anti-PD-1: nivolumab, pembrolizumab; anti-PD-L1: durvalumab, atezolizumab, avelumab) enable the immune system to combat tumor cells, targeting mainly T-cells [1,4]. The study presented in [5] has proved that the efficacy of durvalumab can be potentiated by chemotherapy. Durvalumab has efficacy in advanced urothelial and non-small-cell lung cancers and also suggests benefits in several other tumor types, like cholangiocarcinoma, head, and neck squamous cell carcinoma, tissue and bone sarcoma, and breast carcinoma [6,7,8,9,10]. Developing immune checkpoint inhibitors has led to an important improvement in advanced cancer therapy [11,12]. These therapies are generally better tolerated than chemotherapy. The treatments are associated with diverse adverse events and may initiate distinct autoimmune-related disorders or inflammatory conditions, with different severity, in some cases. Even though these immune-related negative events mainly affect the skin, endocrine glands, digestive tract, heart, joints, liver or lungs, and the hematopoietic system, in theory all organs can be theoretically affected [13]. Cutaneous side effects due to treatment with immune checkpoint inhibitors are usually self-limiting and easily manageable [14]. We present a rare clinical case of a cutaneous side effect due to durvalumab treatment, which was not presented in the scientific literature in any of the databases: Web of Science, Scopus, PubMed and others. We have performed a comprehensive in-depth search including all time publications up to now by verifying combinations of keywords in the whole manuscripts that confirm the originality of our finding.

## 2. Case Report

**Figure 1:**
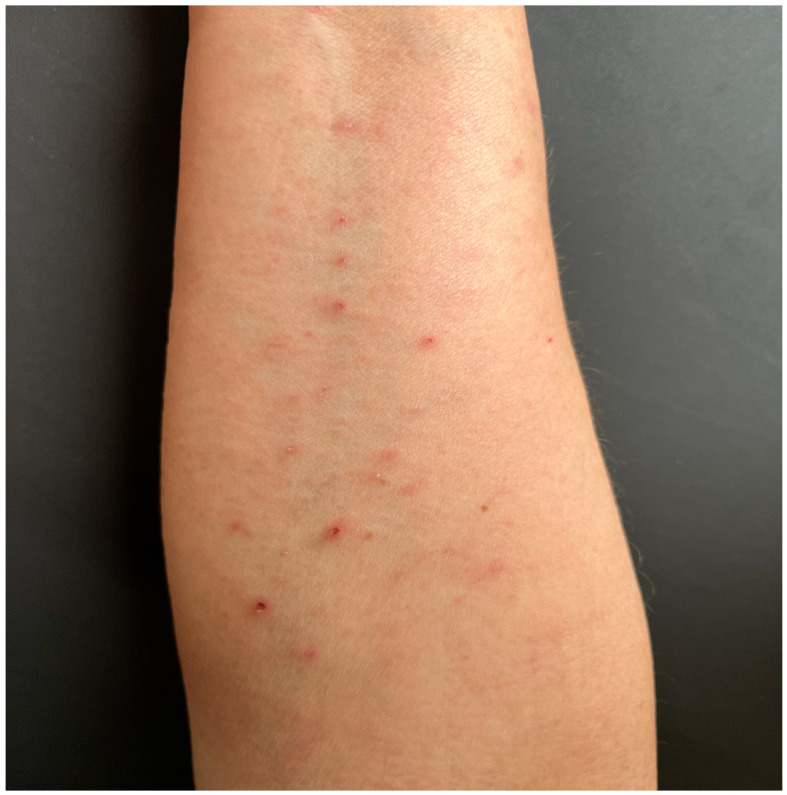
Clinical aspect—Multiple papulo-necrotic lesions on the forearm.

We present a clinical case of a female patient aged 61, who is a smoker, weighing 65 kg, diagnosed in 2021 with small cell lung carcinoma exhibiting mediastinal extension, specifically stage III B, cT3N2M. The diagnosis was established following a CT scan and bronchial mucosa biopsy, conducted subsequent to a prior SARS-CoV-2 infection. The patient initiated a five-cycle treatment comprising Durvalumab DT 1500 mg, Carboplatin AUC5 DT 638 mg, and Etoposide DT 178 mg, demonstrating good tolerability and no acute toxicity. Subsequently, the patient continued with monotherapy using Durvalumab, administered once every four weeks, intravenously for a duration of seven months, showing a favorable response. Shortly thereafter, the patient sought consultation at the Dermatology outpatient unit, due to the emergence of multiple round/oval, papulovesicular, and papule-necrotic lesions dispersed across the lower back, abdomen, upper, and lower limbs, with size between 3 mm and 1 cm, with a total of more than 50 lesions (Figure 1).

**Figure 2:**
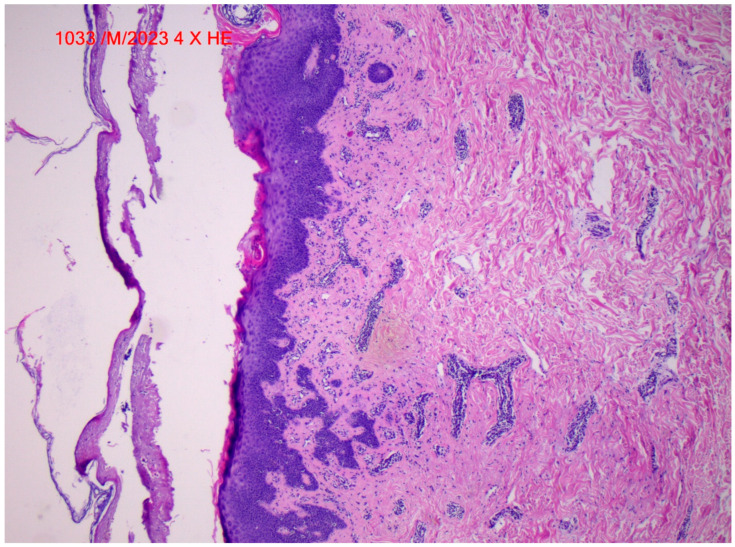
Cutaneous leukocytoclastic vasculitis. HE stains, 4×.

The lesions were associated with moderate pruritus and pain. Moderate xerosis of skin was also present, particularly on the lower limbs’ area. No nail involvement was found. The patient did not feel feverish, nor did they experience abdominal pain, arthralgia or other important subjective symptoms. A suppositional diagnosis of cutaneous vasculitis was made. Laboratory results evidenced only a slight increase in inflammation markers and discrete anemia. The renal function was unimpaired, and the outcome of the urinalysis was within normal range. No other clinical evidence, nor laboratory and imagistic findings possibly related to internal organ involvement were seen. Based on the clinical, paraclinical, and imagistic findings we exclude the large and medium vessel vasculitises. In June 2023, a cutaneous biopsy revealed cutaneous leukocytoclastic vasculitis presenting a dense perivascular neutrophilic infiltration, fibrinoid necrosis of the vessel walls, leukocytoclastic, and red blood cell extravasation, excluding other types of small vessel vasculitis, like Wegener’s granulomtosis, Churg Strauss syndrome, microscopic polyangiitis, Henoch Schönlein purpura or Essential cryoglobulinemic vasculitis (Figure 2). She underwent treatment with methylprednisolone at a dosage of 0.5 mg/kg for three weeks, and locally topical Clobetasol propionate ointment at 0.1% once daily, yielding lesion remission. Importantly, the Durvalumab treatment was not discontinued. After another cycle of Durvalumab, the lesions reappeared. We ruled out alternative reasons for cutaneous vasculitis through clinical and laboratory observations, and so the reappearance of the cutaneous lesions after the treatment with Durvalumab confirmed the etiology. In consultation with the oncologist, we opted to discontinue the Durvalumab treatment and reassess the patient for alternative follow-up therapies.

## 3. Discussion

Immune checkpoint inhibitors are becoming an important drug group in cancer immunotherapy. Their clinical success is based on improving immune suppression and driving durable antitumor T-cell responses. Checkpoint inhibitors inhibit the regulatory interactions that limit T-cell cytotoxicity to tumors. These molecules attack either CTLA-4 or the PD-1/PD-L1 pathways. Because they may also regulate autoreactivity, immune checkpoint inhibitor therapy is made more complex by side effects, known as immune-related adverse events (irAEs) [15]. These toxicities can result in a multitude of side effects, ranging from mild to severe and debilitating, affecting many organs. The mechanism of the appearance of these side effects is still unclear. Das et al. [16]. conclude that there is an association between early therapy-induced changes in circulating B cells and an elevated risk of high-grade immune-related adverse events (IRAEs) in patients with checkpoint inhibitors that target cytotoxic T lymphocyte-associated antigen-4 (CTLA4) and programmed cell death protein 1 (PD1). These findings pinpoint likely predictive biomarkers for high-grade IRAEs that may be used to improve patient monitoring and may help pave the way to new treatment strategies to prevent IRAEs [17]. One of the most important systemic severe side effects on the heart is the appearance of myocarditis. These immune responses in the heart are potentially serious because they have the ability to interfere with the electromechanical function. Moreover, the myocardium has a finite regenerative capacity with which to repair damage caused by effector T-cells. Grabie et al. [18]. conclude in their studies with mice that genetic shortcomings of checkpoint molecules, including PD-1, PD-L1, CTLA-4, and lymphocyte activation gene-3, result in elevated risk of autoimmune T-cell-mediated myocarditis, as well as increased pathogenicity of heart antigen-specific effector T-cells. Many questions about myocarditis in these immunotherapies still need to be clarified. The most frequent endocrine irAEs associated with checkpoint inhibitor treatment are thyroid, adrenal, and pituitary gland dysfunctions. Hypophysitis is in most cases the result of anti-CTLA-4 treatment. Type 1 diabetes mellitus and adrenalitis are both rare side effects. Combination therapy (anti-CTLA-4 plus anti-PD-1/PD-L1) is in some instances related with an increased risk and prevalence of endocrine immune-mediated side effects [1]. Side effects include arthritis and other rheumatic manifestations. The prevalence of irAEs in these cases is related to the inhibited checkpoint. (B5) Michot et al., among the 63 reported cases with hematologic irAEs due to Immune checkpoint inhibitors, found the existence of immune thrombocytopenia (*n* = 18, 29%), pancytopenia or immune aplastic anemia (*n* = 12, 19%), neutropenia (*n* = 11, 17%), hemolytic anemia (*n* = 10, 16%), cytokine release syndrome with hemophagocytic syndrome (*n* = 7, 11%) and other disorders, like bi-cytopenia or pure red cell aplasia (*n* = 5, 8%). These side effects are in most cases extremely severe with a mortality rate as high as 14% (9 deaths among the 63 cases reported). The more serious and life-threatening Hem-irAEs were both cytokine release syndrome with hemophagocytic disorder and pancytopenia or aplastic anemia. In conclusion, hematological severe side effects induced by these drugs are potentially life-threatening [2]. There are published data about the involvement of hepatic, pulmonary and renal organs, due to the side effects of immune checkpoint inhibitor treatments [19,20,21]. Rarely, colitis, infections like meningitis, central and peripheral nervous system problems, and severe allergic reactions can also be present [22]. As a consequence of their severity, irAEs can limit therapy and require immunosuppression therapy. Rare severe side effects, like Stevens-Johnson syndrome and other severe cutaneous immune-related adverse events, require inpatient care as well as urgent dermatology examination and specific treatment [11,23]. In addition to the side effects already described, Collins et al. [24] describe the appearance of systemic lupus and sarcoidosis. Sweet syndrome and severe cutaneous toxicities were reported, like toxic epidermal necrolysis and severe drug rash, with eosinophila and systemic symptoms. The authors conclude that cutaneous events usually occur early in the course of treatment and are dose-dependent [24]. Several cutaneous side effects regarding the immune checkpoint inhibitors are described in the literature. Plachouri et al. [25]., in an overview of the most frequent immune-mediated cutaneous side effects of immune checkpoint inhibitors, stipulated that the most common adverse cutaneous reactions include maculopapular rash, lichenoid reactions, vitiligo, and pruritus, with a milder severity grade. Rarer but sometimes life-threatening side effects of the skin, including Stevens–Johnson syndrome, drug reaction with eosinophilia, and toxic epidermal necrolysis, have also been noted. The most frequent skin side effects, as mentioned above, should be receive symptomatic treatment, so that permanent discontinuation of immunotherapy is not required. The authors suggest that, in the case of life-threatening side effects, the immunotherapy should be permanently stopped, and it is necessary to start symptomatic treatment. Some authors even described lichenoid oral mucosal lesions, dermatomyositis, and lupus erythematosus. Muntyanu et al. [26]. conclude that the management of cutaneous severe side effects of immune therapies may encourage clinicians to reduce dosages, add systemic steroids to the treatment regimen, and/or discontinue immunotherapy. The goal, therefore, is for early recognition of these cutaneous side effects to limit treatment interruptions. They also suggest that the severity of the reaction should not be categorized based solely on body surface area involvement, but instead on the clinical aspect of cutaneous pathology. For example, maculopapular outbreaks rarely affect <30% BSA and can often be managed locally with skin-directed therapies, while Stevens-Johnson syndrome (SJS) affecting even 5% BSA is better treated systemically, including stopping immunotherapy. There are, at present, very few scientific studies related to the management of the cutaneous severe side effects and most of those present largely anecdotal evidence. The authors also reviewed the management strategies and provided recommendations for psoriatic, immuno-bullous, maculopapular, lichenoid, acantholytic eruptions, vitiligo, alopecia, vasculitides, SJS/toxic epidermal necrolysis, and other associated skin conditions. Some life-threatening diseases, like Steven Johnson and toxic epidermal necrolysis, were reported. Geisler et al. [23] conclude that the most prevalent cutaneous immune-related detrimental events include a wide variety of inflammatory reactions, with maculopapular rash, pruritus, psoriasiform and lichenoid eruptions. Cutaneous immune-related adverse events present themselves early on, with maculopapular rash occurring within the first 6 weeks after the first immune checkpoint inhibitor dose. In general, the authors recommend that the treatment of mild to moderate rashes due to these side effects involves the use of topical corticosteroids. For severe rashes, the addition of systemic corticosteroids and the discontinuation of immunotherapy is prescribed. In addition, bullous pemphigoid eruptions, vitiligo-like skin hypopigmentation/depigmentation, and psoriasiform rash can occur separately. These should be treated differently. For instance, the treatment of bullous pemphigoid eruptions is similar to the treatment of maculopapular rash and lichenoid eruptions, with the addition of rituximab in grade 3–4 rash. Vitiligo like lesions do not require specific dermatologic treatment, but only photo-protective measures. In addition to topical corticosteroids, psoriasiform type lesions can be treated with vitamin D3 analogues, narrowband ultraviolet B light phototherapy, retinoids, or immunomodulatory biologic agents. Sibaud et al. [27] present a wide range of dermatologic manifestations that can occur as side effect of checkpoint inhibitors immunotherapy, including rare clinical diseases, like lichenoid reactions, psoriasis, acneiform rashes, sicca syndrome, autoimmune skin disorders, which include (e.g., bullous pemphigoid, dermatomyositis, alopecia areata), sarcoidosis, or nail and oral mucosal changes. Bhardwaj et al. [28], in an overview regarding cutaneous irAEs, conclude that recognition of cutaneous involvement at early stages could help in better management and would prevent treatment discontinuation. The authors highlight that cutaneous adverse effects are the most prevalent immune-related adverse events induced by immune checkpoint inhibitors. They summarized the clinical forms they have found, similar to the cited authors below. Nikolaou et al. [29]. in a multicenter retrospective international cohort study of cancer patients who developed cutaneous irAEs under ICI therapy, including 762 patients who developed 993 cutaneous toxicities, concluded that the most frequent skin diseases in descending order were: psoriasis (175 patients, 23.0%) followed by (171 patients, 22.4%), macular rash (161 patients, 21.1%) and eczematous-type reactions (150 patients, 19.7%). This study showed that ICI-related skin toxicities do not share a single pattern, but rather likely depend on a number of factors, particularly the drug employed and the present malignancy type [29]. Another study performed in the USA by Wongvibulsin et al. [30], including 8637 ICI patients, shows that the overall incidence of cutaneous irAEs was 25.1%, with a median onset time of 113 days. This ICI group compared with the same number in a control group had a significantly higher incidence of pruritus, mucositis, erythroderma, maculopapular eruption, vitiligo, lichen planus, bullous pemphigoid, Grover disease, rash, other nonspecific eruptions, and drug eruptions, or other nonspecific drug reactions. Patients with melanoma and renal cell carcinoma and those receiving combination therapy were at a higher risk of having cutaneous side effects [30]. The most frequent skin side effects tend to respond to symptomatic treatment, making the permanent discontinuation of therapy unnecessary. On the contrary, in the event of severe or life-threatening side effects, in addition to the necessary symptomatic treatment, the immunotherapy should be stopped, as in our case. 

As we found in the literature, there was only one case of cutaneous vasculitis described in association with the chronic use of Durvalumab, more specifically the blue toe syndrome, a more localized clinical form [31]. In our case, we present the association between Durvalumab and a generalized form of cutaneous vasculitis. To the best of our knowledge, our case is the first published case of disseminated cutaneous vasculitis as a result of Durvalumab treatment. In Table 1, we present the published side effects caused by immune checkpoint inhibitor treatment.

In the searched databases, we did not find any cases presenting cutaneous vasculitis as a side effect, due to other immune checkpoint inhibitors, like Nivolumab, Pembrolizumab, Atezolizumab, etc. Cutaneous vasculitis as a side effect has been documented in the context of other immunotherapies used in advanced lung cancers, such as Sorafenib or Erlotinib [32,33]. Fekete et al. [34]. concluded that the emergence of cutaneous vasculitis due to erlotinib treatment might serve as an exacerbating factor in the progression of advanced lung cancer. For drawing of insightful conclusions about cancer prognosis due to cutaneous vasculitis, frequent dermatological evaluation is thus mandatory in the case of atypical, persistent/recurrent or severe cutaneous lesions due to these therapies.

### Challenges and Prospective Future Application of Machine Learning

Artificial intelligence (AI), generally Machine Learning (ML), recently has experienced many strong developments in health sciences [35]. Machine Learning could offer diverse types of decision support in the field of dermatology, like predictions and prognostics. Decision support could be useful for physicians, including dermatologists, in making more accurate decisions in elaboration of diagnostics and treatments [36]. 

We have not found any research that could be related to our finding presented in the case study and, based on this fact, we consider that this it as an open research direction. In the following, some related publications are presented regarding diverse developments that could be further extended for decision support in treating Disseminate cutaneous vasculitis cases. 

Jutzi et al. [37]. studied the application of AI for skin cancer diagnostics. For addressing the suitability there are studied the patients’ concerns and questions using a survey. The scope of the survey consisted in the evaluation of the patients’ view on AI in the diagnostics of melanoma. For the results evaluation there were performed statistical tests being investigated associations between selected items of the questionnaire and collected sociodemographic data. Questioning was performed on 298 people, of which 143 were not diagnosed with melanoma and 154 with a melanoma diagnosis. Most of the questioned persons were optimistic toward the use of AI in melanoma diagnostics.

Wei et al. [36]. presents a recent comprehensive review on diverse applications of AI including machine learning (ML) that could offer support to dermatologists and dermatopathologists in decisions elaboration regarding diagnosis and treatment plans. The applications consist in the fields of skin cancer diagnosis and screening. There are presented approaches based on technologies like assistive and disruptive. Treated models are applied for molecular and image processing. At the same time are presented diverse challenges to clinical implementation, paths forward for implementation. The review paper suggests many promising future perspectives of research regarding the application in the field of dermatology. 

Sanchez et al. [38]. presents some recent applications of AI for non-melanoma skin cancer detection. Much AI research related to dermatology focuses on melanoma. The study of prevalence of non-melanoma skin cancers is less approached. For example, can be mentioned the basal cell and squamous cell cancer [38]. is focused on decision support care in the absence of dermatology expertise. One of the applications in this sense is predicting therapeutic response and assists in designing novel therapeutics. In this research, the important subject is outlined of the necessary data and information that should be used to train the models. In this context, dermatologists have an important role in curating high-quality datasets along with methods used for data preprocessing. Performance of models is also a key for promoting confidence in newly developed systems. AI solutions will not replace the dermatologist, but they can be used as a complementary tool in decision making.

Hekler et al. [39]. present a study of patients’ perspective on AI for skin cancer analysis. Human–machine reasoning on images for detection and classification of skin cancer is proposed. Research results obtained prove that this methodology resulted in an increased accuracy of 82.95% compared to studied artificial intelligence models and humans individually. The development of novel ML models is highly dynamic, obtaining more and more accurate results, but we are convinced that combination of human and artificial reasoning is beneficial. AI notices could be considered by physicians in decisions but the final diagnosis and treatment decisions that are effectively applied could be made by human physicians. 

In our previous research [40], similarly to the study presented in [39], we proposed the theoretical foundations of complex combined human and AI reasoning by teams of physicians for highly complex medical diagnostics elaboration that can be applied even for complex and rare diagnosis cases. The proposed so-called Intelligent Medical Hybrid System (IntHybMediSys), which is an agent-based system, assists in collaboration with a physician team and offers intelligent decision support. The developed reasoning represents an extension of the so-called blackboard-based problem solving proposed in the field of artificial intelligence.

Based on our previous research and the performed bibliographic study, we emphasize the importance of the application of ML models, even for very rare case analyses. Difficulties in such situations are based on data on very few cases, which could bring difficulties in developing accurate models. Physicians also could show limitations in the formulation of pertinent conclusions based on very little data. For such situations, we consider appropriate AI solutions that provide support in collaboration for such analyses, and support in decision making by performing different background computations. The report in [41] presents an in-depth study of the cognitive complexity of designing the group decision process. Proceedings for decision support can be very diverse and should be based on very diverse methods of artificial intelligence, ranging from rule-based systems to natural language processing of manuscripts from the scientific literature to large language models and transformers. Another aspect that is also very rarely treated is the model explainability. It could be useful to physicians if AI provides not only suggestions, as for prediction and diagnosis, but also gives explanations.

## 4. Conclusions

The patient is currently undergoing both dermatologic and oncologic follow-up, and further assessment is needed to determine appropriate treatment options. In conclusion, we find that thorough clinical examination of the skin at regular intervals is mandatory for all patients, regardless of the cancer type, for the timely diagnosis of possible cutan involvement in oncologic treatments, as well as for identifying cutan metastasis and establishing optimal treatments for associated skin conditions in oncologic patients. Long-term management of our case involves a multidisciplinary approach to balance cancer and to diagnose and treat cutaneous side effects of oncologic treatment. Hence, periodic clinical examination of the skin, blood tests for markers of inflammation, and skin biopsy when needed, must be carried out.

In this context, the usefulness of artificial intelligence-based decision support systems can be outlined, which can offer support to medical doctors in different points of decision making. This could have diverse benefits, like decreasing human effort and increasing diagnostic and prognostic accuracy. We have presented some challenges regarding AI and future perspectives in the combination of human and Artificial Intelligence-based reasoning for studying the Disseminate cutaneous vasculitis associated with Durvalumab treatment.

## Figures and Tables

**Table 1:** Published cutaneous side effects of immune checkpoint inhibitors.

Cutaneous Side Effects of Immune Checkpoint Inhibitors	
Mild	Severe
Pruritus Cutan xerosis Eczemas Maculo-papular rash Lichenoid reactions Oral mucosal lesions Drug rash with eosinophilia Acneiform rashes Alopecia areata Localized cutan vasculitis	Severe drug rash Steven Johnson syndrome Toxic epidermal necrolysis Dermatomyositis Sweet syndrome Sicca syndrome Lupus erythematosus Bullosus pemphigoid Sarcoidosis Disseminate cutaneous vasculitis

## Data Availability

Dataset available on request from the authors.
